# Supplementary material for: Synthetic cytokine receptors transmit biological signals using artificial ligands
Source: Nat Commun. 2018 May 23;9:2034. doi: 10.1038/s41467-018-04454-8 (PMC5964073; doi:10.1038/s41467-018-04454-8)
Supplement: Supplementary file 1 — Supplementary Information [file 41467_2018_4454_MOESM1_ESM.pdf]

Supplementary Figure 1

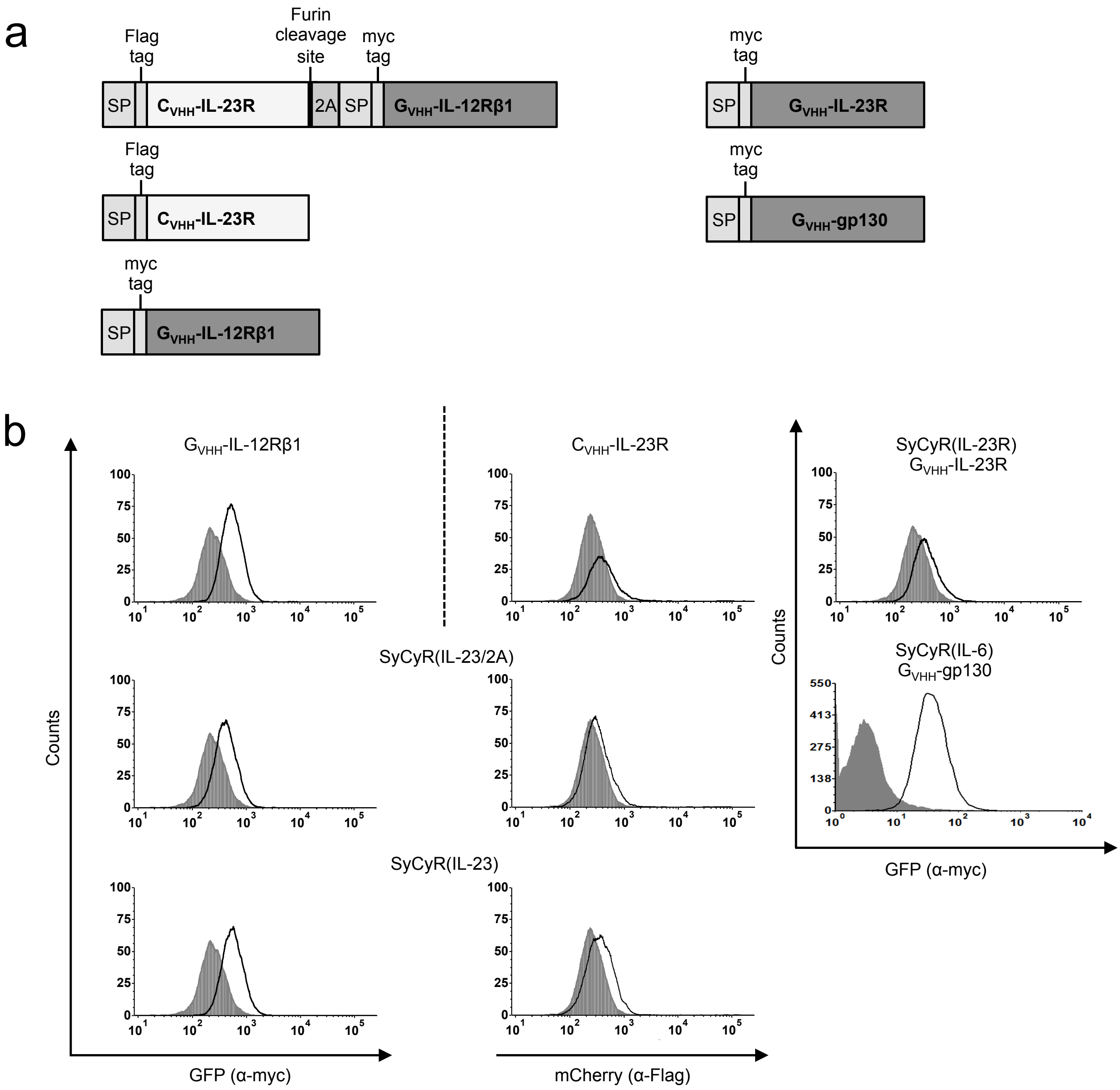

Supplementary Fig. 1 Expression cassettes and cell surface expression of SyCyRs. **a** The expression cassettes for all SyCyRs consist of a signal peptide from IL-11R followed by a Flag- or myc-tag and the C_{VHH} or G_{VHH}, 13-17 aa of the extracellular part, the transmembrane and intracellular domains of the cytokine receptor. **b** Cell surface expression of SyCyRs (light solid lines). Indicated are only the transduced SyCyRs in Ba/F3-gp130 cells, detected by myc- or Flag-antibodies. Gray-shaded areas indicate Ba/F3-gp130 cells (negative control).

Supplementary Figure 2

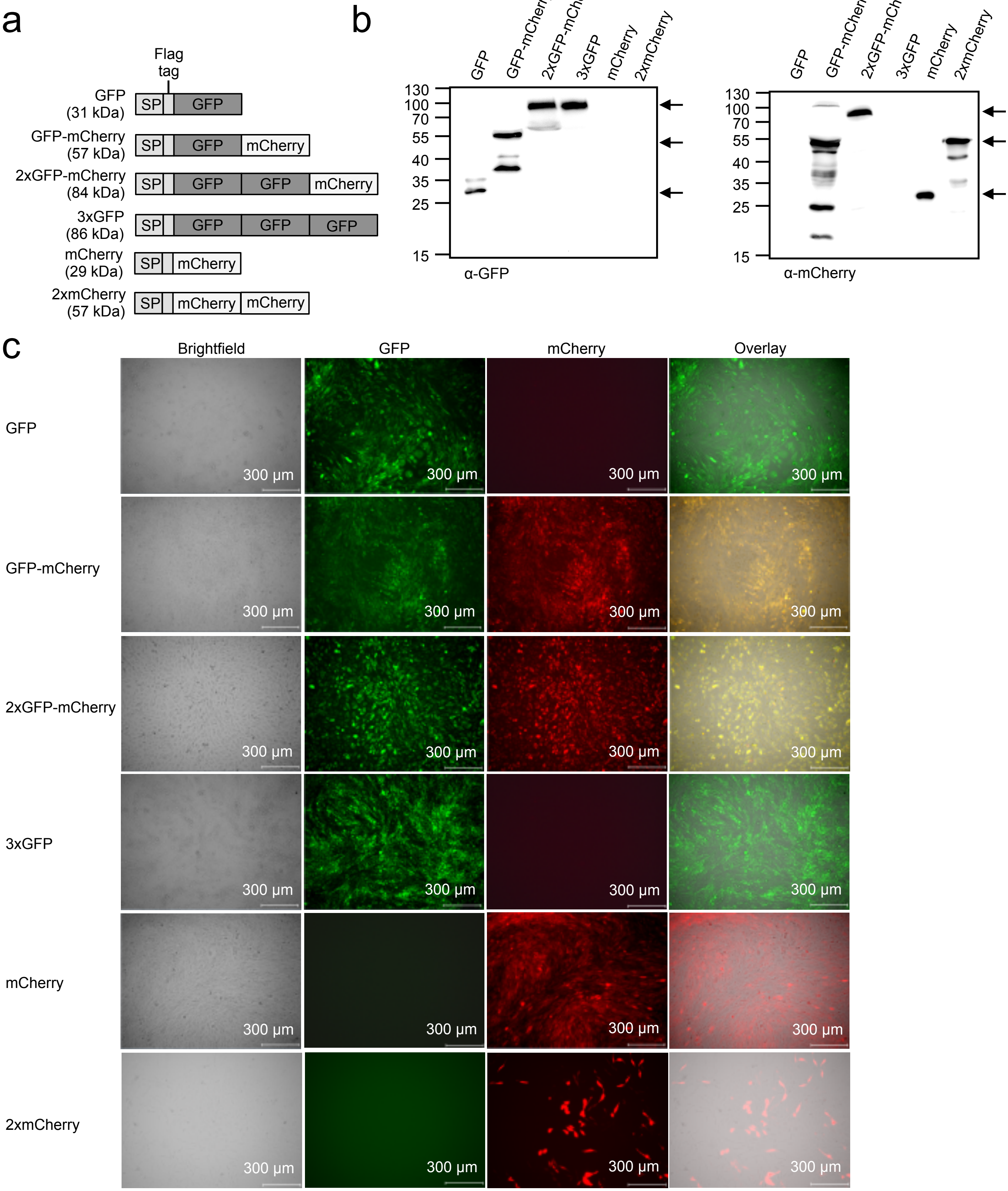

Supplementary Fig. 2 Expression cassettes and cellular expression of GFP, mCherry and fusions of these. **a** The expression cassettes for GFP, mCherry and fusions thereof consist of a signal peptide from IL-11R followed by a Flag-tag and the GFP or mCherry coding sequences. **b** Western blotting of all GFP, mCherry and fusions thereof secreted from stably transduced CHO-K1 cells using GFP and mCherry mAbs. **c** Stably transduced CHO-K1 cells exhibited green or red fluorescence due to the expression of GFP, mCherry and fusions thereof.

Supplementary Figure 3

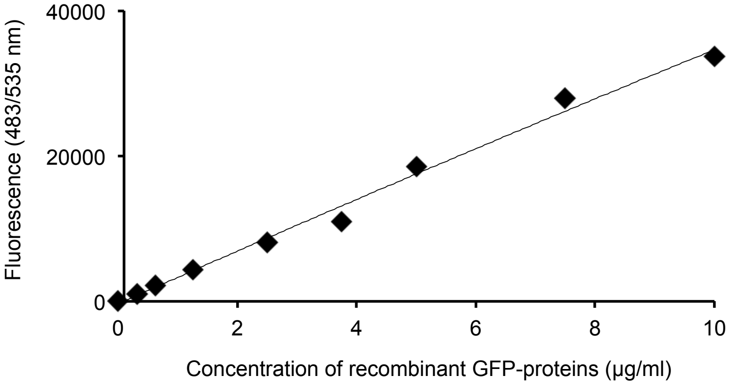

	Fluorescence	Concentration (µg/ml)
**3xGFP**	9427	2.71
**2xGFP-mCherry**	10175	2.93
**GFP-mCherry**	14020	4.03
**GFP**	15281	4.39

Supplementary Fig. 3 Quantification of GFP and GFP:mCherry fusion proteins in cell culture supernatants. Conditioned cell culture supernatants from stably transfected CHO-K1 cells expressing GFP and GFP:mCherry fusion proteins were quantified by fluorescence measurement with an excitation maximum at 483 nm and fluorescence emission at 535 nm. Provided that the fluorescence of GFP and its variants correlates positively with their concentration, recombinant GFP was used to create a calibration curve. Recombinant GFP was diluted from 10 to 0.3 µg/ml in cell culture supernatants from untransfected CHO-K1 cells which were treated the same way as stably transfected CHO-K1 cells. All fluorescence values were measured in duplicate and normalized by subtraction of values of supernatants from untransfected CHO-K1 cells.

Supplementary Figure 4

a

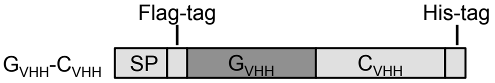

b

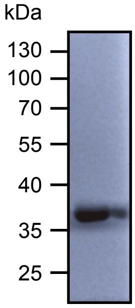

Supplementary Fig. 4 Recombinant G_{VHH}-C_{VHH} purified from *E.coli*. **a** The expression cassettes of the G_{VHH}-C_{VHH} fusion protein consist of a PelB signal peptide followed by a Flag-tag, G_{VHH}, C_{VHH} and a His tag. **b** Purified G_{VHH}-C_{VHH} (5 μg) was separated on a SDS-PAGE gel and stained by Coomassie brilliant blue.

Supplementary Figure 5

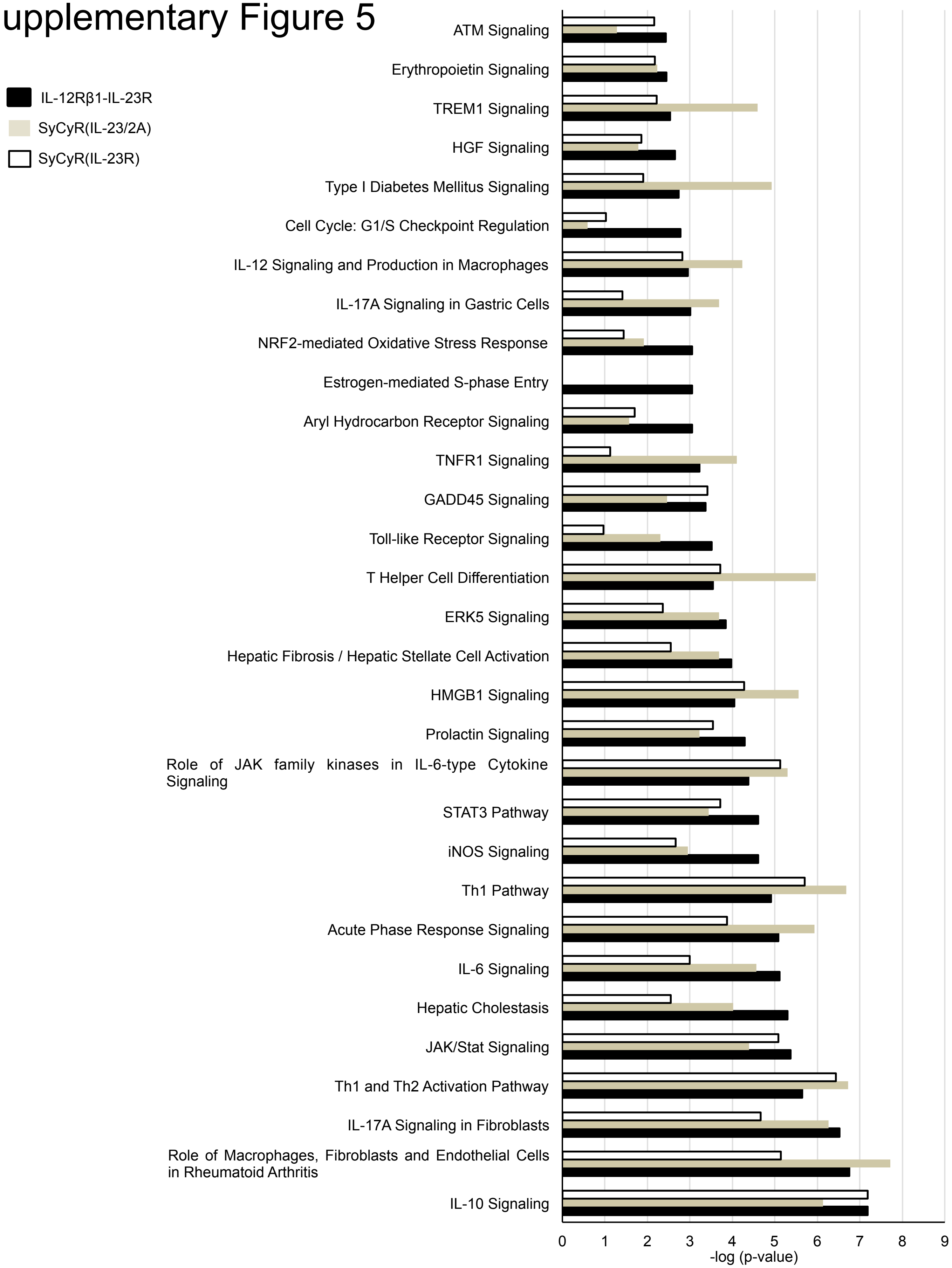

Supplementary Fig. 5 The top canonical pathways of Ba/F3-IL-12Rβ1-IL-23R compared to Ba/F3-SyCyR(IL-23/2A) and Ba/F3-SyCyR(IL-23R). The diagram shows if the top 30 canonical pathways of Ba/F3-IL-12Rβ1-IL-23R could also be identified for Ba/F3-SyCyR(IL-23/2A) and Ba/F3-SyCyR(IL-23R) cells.

Supplementary Figure 6

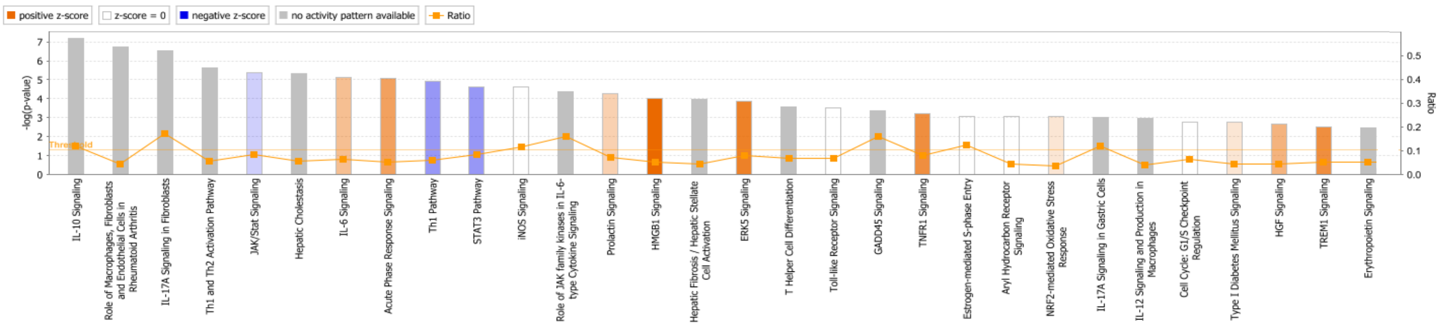

Molecular and Cellular Function of IL-12Rβ1-IL-23R

Name	P-value	#Molecules
Cellular Development	4.10E-06 – 9.80E-20	94
Cellular Growth and Proliferation	4.10E-06 – 9.80E-20	87
Cellular Function and Maintenance	1.11E-06 – 1.33E-19	60
Gene Expression	3.54E-06 – 7.10E-17	76
Cell Death and Survival	4.50E-06 – 1.49E-16	81

Top Analysis-Ready Molecules of IL-12Rβ1-IL-23R

Expr. Fold Change up-regulated

Molecules	Expr. Value
IL-10	39.83
Socs3	23.73
Osm	21.74
ZFP36	16.48
EGR2	15.25
ADORA3	14.41
Gadd45g	12.59
IER3	12.31
LIF	9.70
IL-6	9.22

Supplementary Fig. 6 Analysis of pathways and molecular functions of Ba/F3-IL-12Rβ1-IL-23R. The top canonical pathways of Ba/F3-IL-12Rβ1-IL-23R identified by Ingenuity Pathway Analysis (IPA). The table on the left shows the most significant molecular and cellular functions of Ba/F3-IL-12Rβ1-IL-23R identified by IPA. The table on the right shows the top Analysis-Ready Molecules of Ba/F3-IL-12Rβ1-IL-23R identified by IPA.

Supplementary Figure 7

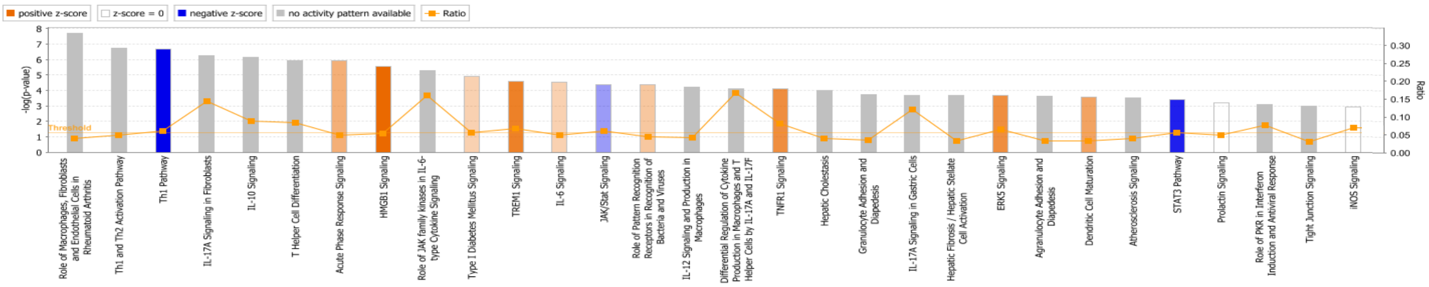

Molecular and Cellular Function of SyCyR(IL-23/2A)

Name	P-value	#Molecules
Cellular Development	2.90E-06 – 2.88E-24	71
Cellular Growth and Proliferation	2.03E-06 – 2.88E-24	67
Cellular Function and Maintenance	1.62E-06 – 4.07E-23	48
Cell Death and Survival	3.07E-06 – 8.67E-17	61
Gene Expression	3.06E-06 – 5.04E-15	51

Top Analysis-Ready Molecules of SyCyR(IL-23/2A)

Expr. Fold Change up-regulated

Molecules	Expr. Value
Osm	14.69
Socs3	13.22
ZFP36	11.03
EGR2	9.46
IL-10	8.97
Gadd45g	8.51
IER3	7.08
LIF	6.64
Pim1	5.92
ADORA3	5.69

Supplementary Fig. 7 Analysis of pathways and molecular functions of Ba/F3-SyCyR(IL-23/2A). The top canonical pathways of Ba/F3-SyCyR(IL-23/2A) identified by Ingenuity Pathway Analysis (IPA). The first table on the left shows the most significant molecular and cellular functions of Ba/F3-SyCyR(IL-23/2A) identified by IPA. On the right the top Analysis-Ready Molecules of Ba/F3-SyCyR(IL-23/2A) identified by IPA is shown.

Supplementary Figure 8

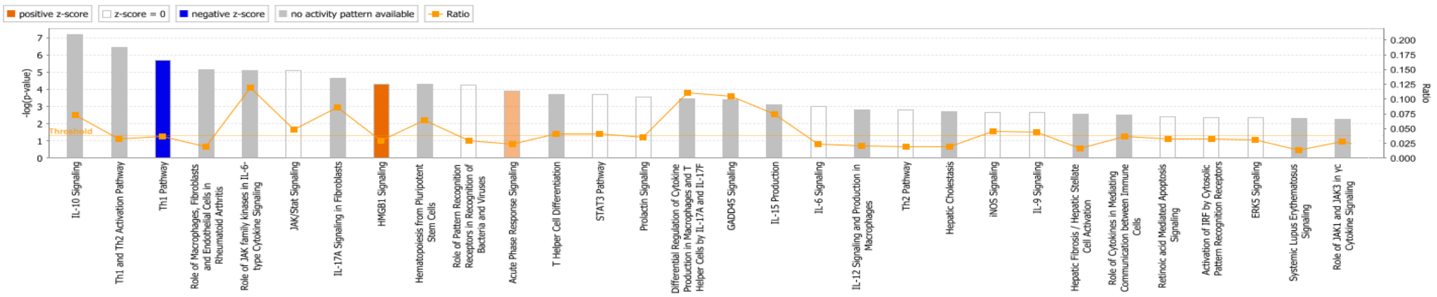

Molecular and Cellular Function of SyCyR(IL-23R)

Name	P-value	#Molecules
Cellular Development	8.77E-05 – 5.72E-18	29
Cellular Growth and Proliferation	8.77E-05 – 5.72E-18	27
Cellular Function and Maintenance	7.58E-05 – 2.63E-16	22
Cell Cycle	5.99E-05 – 2.77E-14	22
Gene Expression	8.48E-05 – 2.77E-14	25

Top Analysis-Ready Molecules of SyCyR(IL-23R)

Expr. Fold Change up-regulated

Molecules	Expr. Value
Socs3	8.39
ZFP36	5.33
Osm	5.22
Gadd45g	4.86
Pim1	4.07
EGR2	3.89
IER3	3.78
LIF	3.07
CDKN2D	2.94
IL-4R	2.78

Supplementary Fig. 8 Analysis of pathways and molecular functions of Ba/F3-SyCyR(IL-23R). The top canonical pathways of Ba/F3-SyCyR(IL-23R). identified by Ingenuity Pathway Analysis (IPA). The table on the left shows the most significant molecular and cellular functions of Ba/F3-SyCyR(IL-23R) identified by IPA. The table on the right shows the top Analysis-Ready Molecules of Ba/F3-SyCyR(IL-23R) identified by IPA.

Supplementary Figure 9

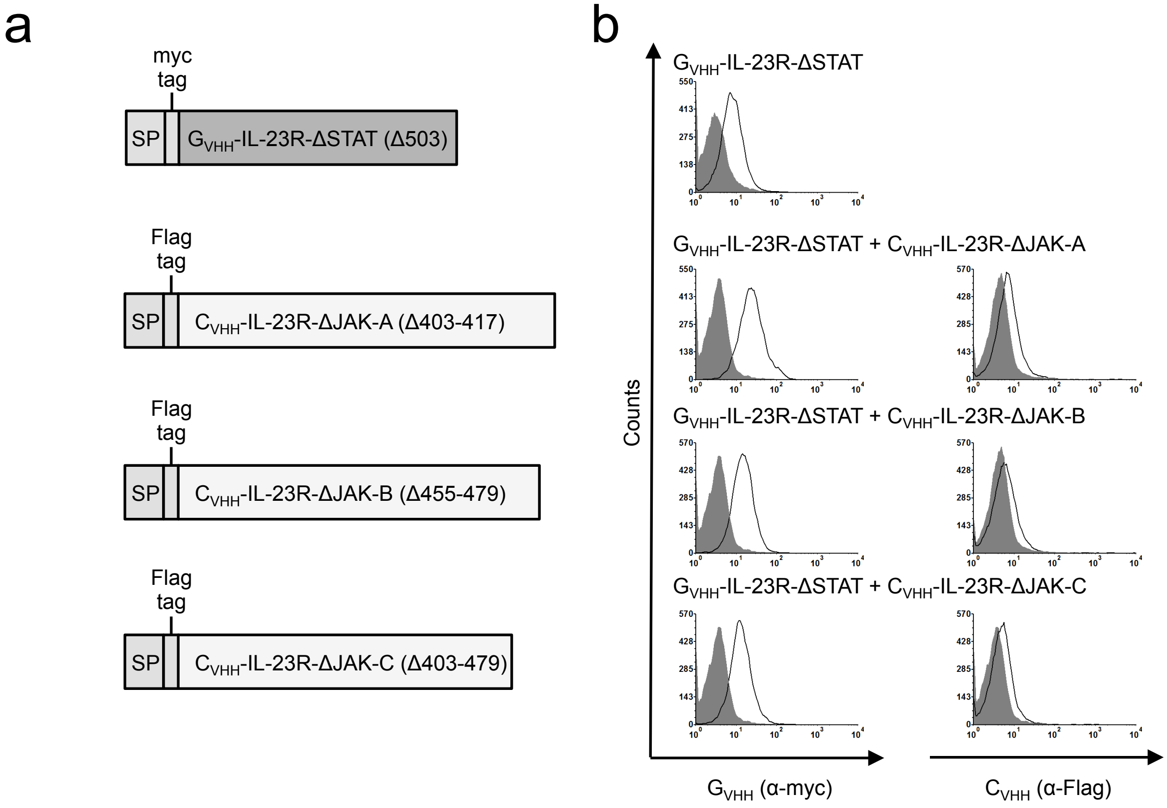

Supplementary Fig. 9 Expression cassettes and cell surface expression of trans-phosphorylation SyCyRs. **a** The expression cassettes for all trans-activation SyCyRs consist of a signal peptide from IL-11R followed by a Flag- or myc-tag and the C_{VHH} or G_{VHH}, 16 to 17 aa of the extracellular part, the transmembrane and intracellular domains of the cytokine receptor. **b** Cell surface expression of trans-phosphorylation SyCyRs (light solid lines). Indicated are only the transduced SyCyRs in Ba/F3-gp130 cells, detected by myc or Flag antibodies. Gray-shaded areas indicate Ba/F3-gp130 cells (negative control).

Supplementary Figure 10

a

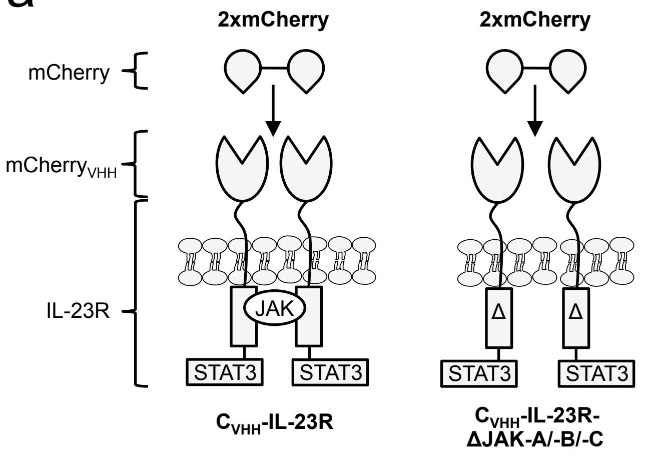

b

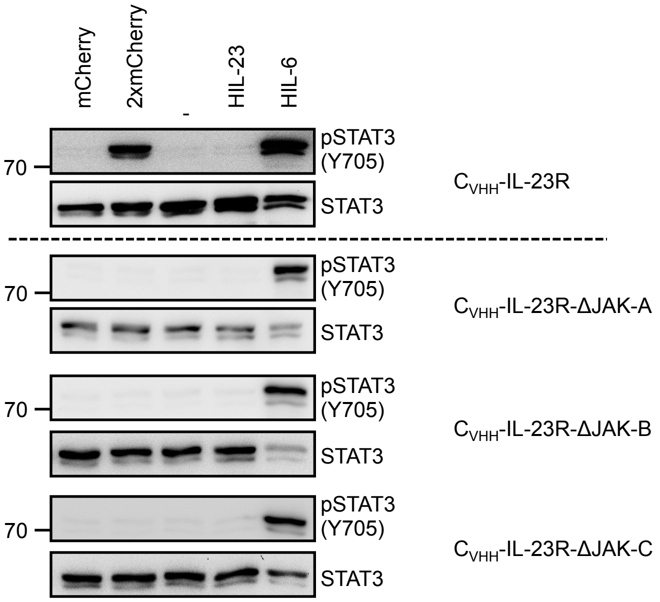

Supplementary Fig. 10 Homodimeric JAK-defective SyCyRs have no STAT3 phosphorylation activity. **a** Schematic illustration of SyCyRs without JAK activity. The mCherry-mCherry fusion protein served as synthetic cytokine ligand. C_{VHH}-IL-23R-ΔJAK variants consist of the C_{VHH} fused to 16-17 aa of the extracellular part, the transmembrane and intracellular domains of the IL-23R lacking JAK activation site (ΔJAK-A,-B,-C). **b** Analysis of STAT3 activation in Ba/F3-C_{VHH}-IL-23R cells and Ba/F3-C_{VHH}-IL-23R-ΔJAK variants. The cells were washed three times, starved and stimulated with 8% CHO-K1 conditioned supernatant containing mCherry and 2xmCherry for 60 min. Stimulation with HIL-23 (10 ng/ml) for 60 min and HIL-6 (10 ng/ml) for 15 min was used as control. Cellular lysates were prepared, and equal amounts of total protein (25 µg/lane for HIL-6 and 50 µg/lane for other ligands) were loaded on SDS gels, followed by immunoblotting using specific antibodies for phospho-STAT3 and STAT3. Western blot data show one representative experiment out of two.

Supplementary Figure 11

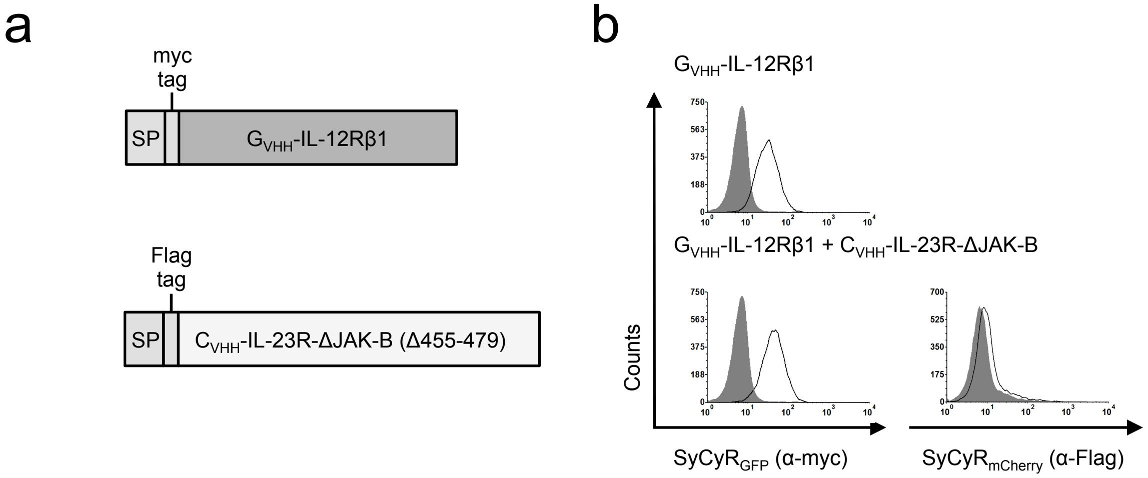

Supplementary Fig. 11 Expression cassettes and cell surface expression of trans-phosphorylation negative SyCyRs. **a** The expression cassettes for all SyCyRs consist of a signal peptide from IL-11R followed by a Flag- or myc-tag and the C_{VHH} or G_{VHH}, 15-17 aa of the extracellular part, the transmembrane and intracellular domains of the cytokine receptor. **b** Cell surface expression of SyCyRs (light solid lines). Indicated are only the transduced SyCyRs in Ba/F3-gp130 cells, detected by myc- or Flag-antibodies. Gray-shaded areas indicate Ba/F3-gp130 cells (negative control).

Supplementary Figure 12

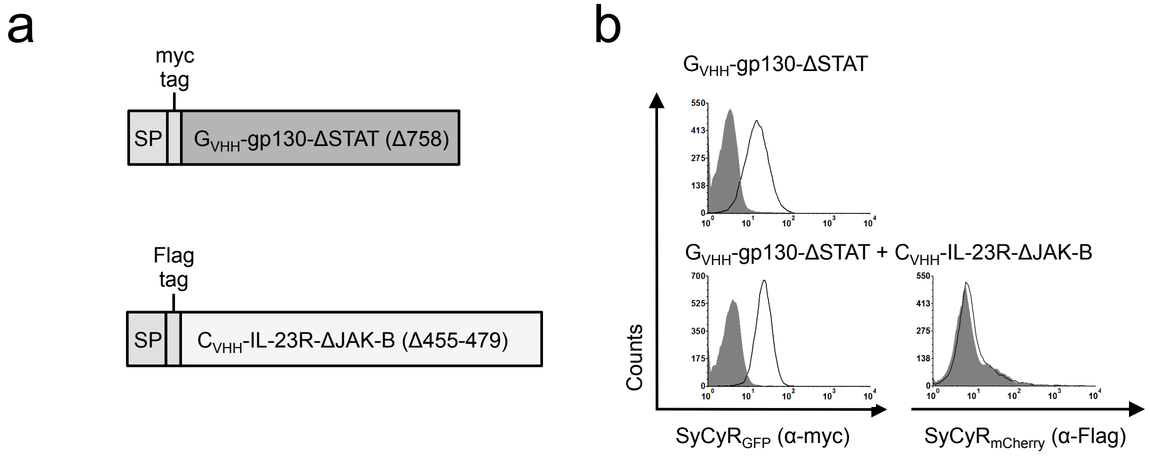

Supplementary Fig. 12 Expression cassettes and cell surface expression of gp130 variants on Ba/F3-gp130 cells. **a** The expression cassettes of G_{VHH}-gp130ΔSTAT(Δ758) in Ba/F3-gp130 cells and the corresponding co-receptors. All SyCyRs consist of a signal peptide from IL-11R followed by a Flag- or myc-tag and the C_{VHH} or G_{VHH}, 13-17 aa of the extracellular part, the transmembrane and intracellular domains of the cytokine receptor. **b** Cell surface expression of G_{VHH}-gp130-ΔSTAT(Δ758) in Ba/F3-gp130 cells and the corresponding co-receptors (light solid lines). Receptors were detected by myc- or Flag-antibodies. Gray-shaded areas indicate Ba/F3-gp130 cells (negative control).

Supplementary Figure 13

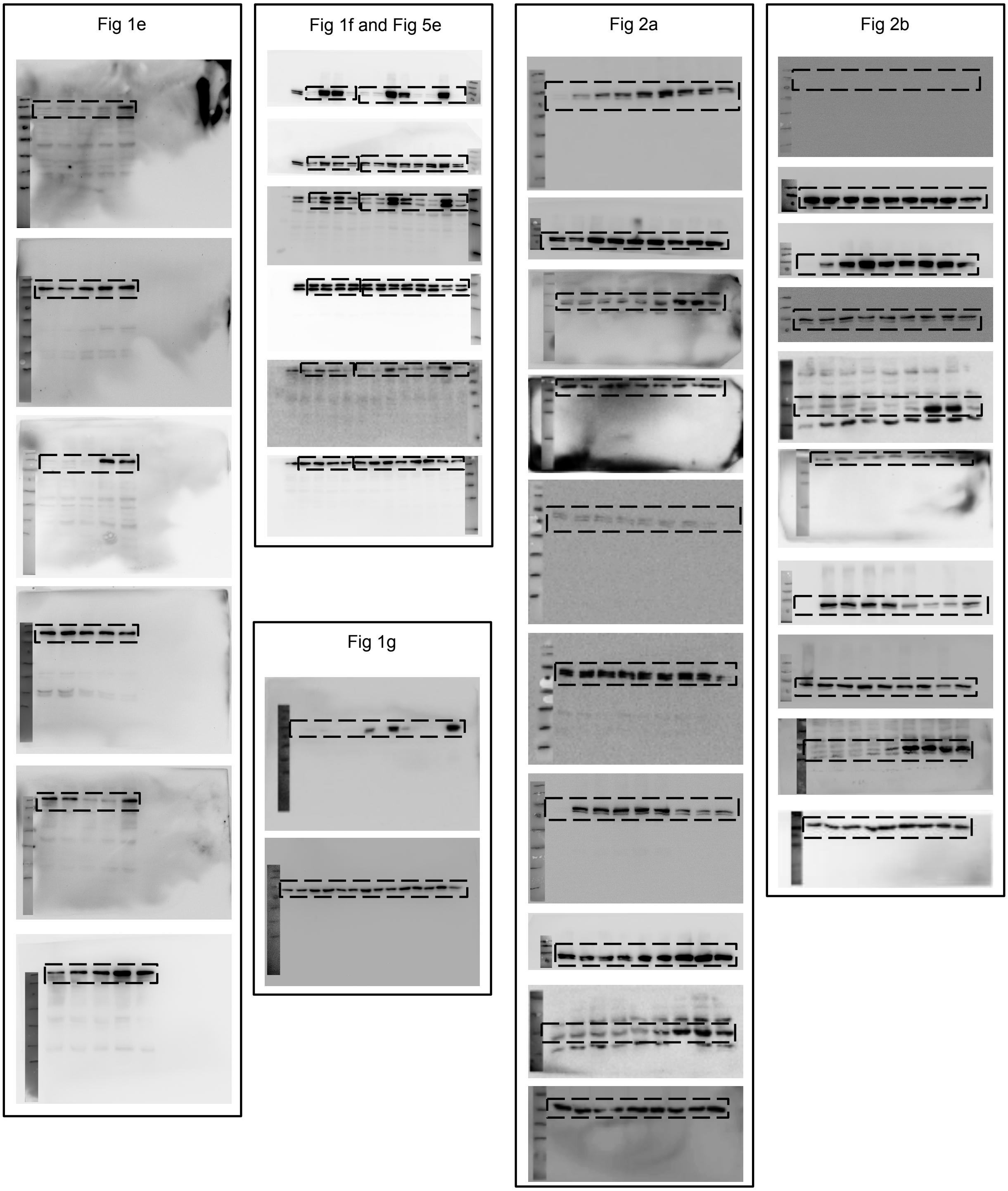

Supplementary Figure 13
Uncropped images of western blots presented in the main manuscript. Black dotted box indicates areas that were cropped.

Supplementary Figure 13

Fig 4b

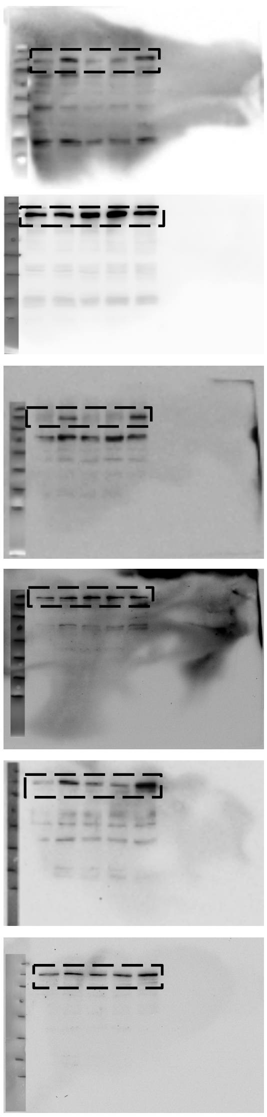

Fig 4c

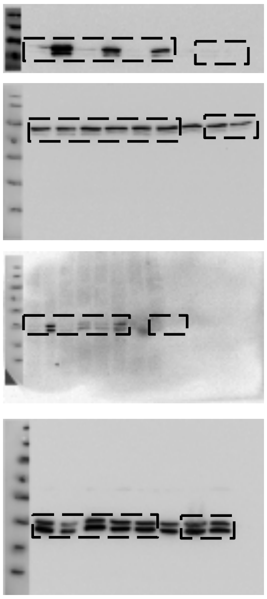

Fig 4e

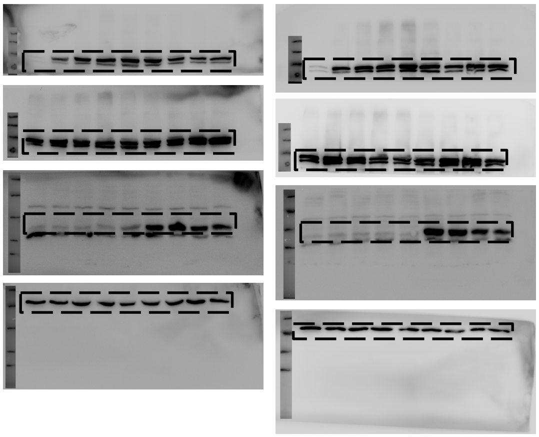

Fig 4f

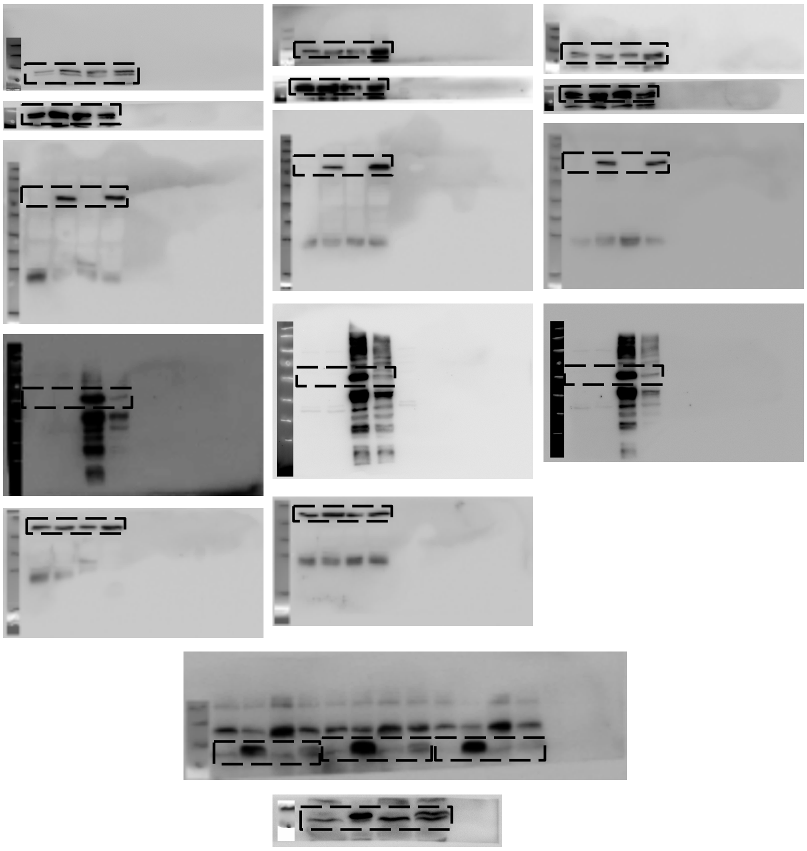

Supplementary Figure 13
Uncropped images of western blots presented in the main manuscript. Black dotted box indicates areas that were cropped.

Supplementary Figure 13

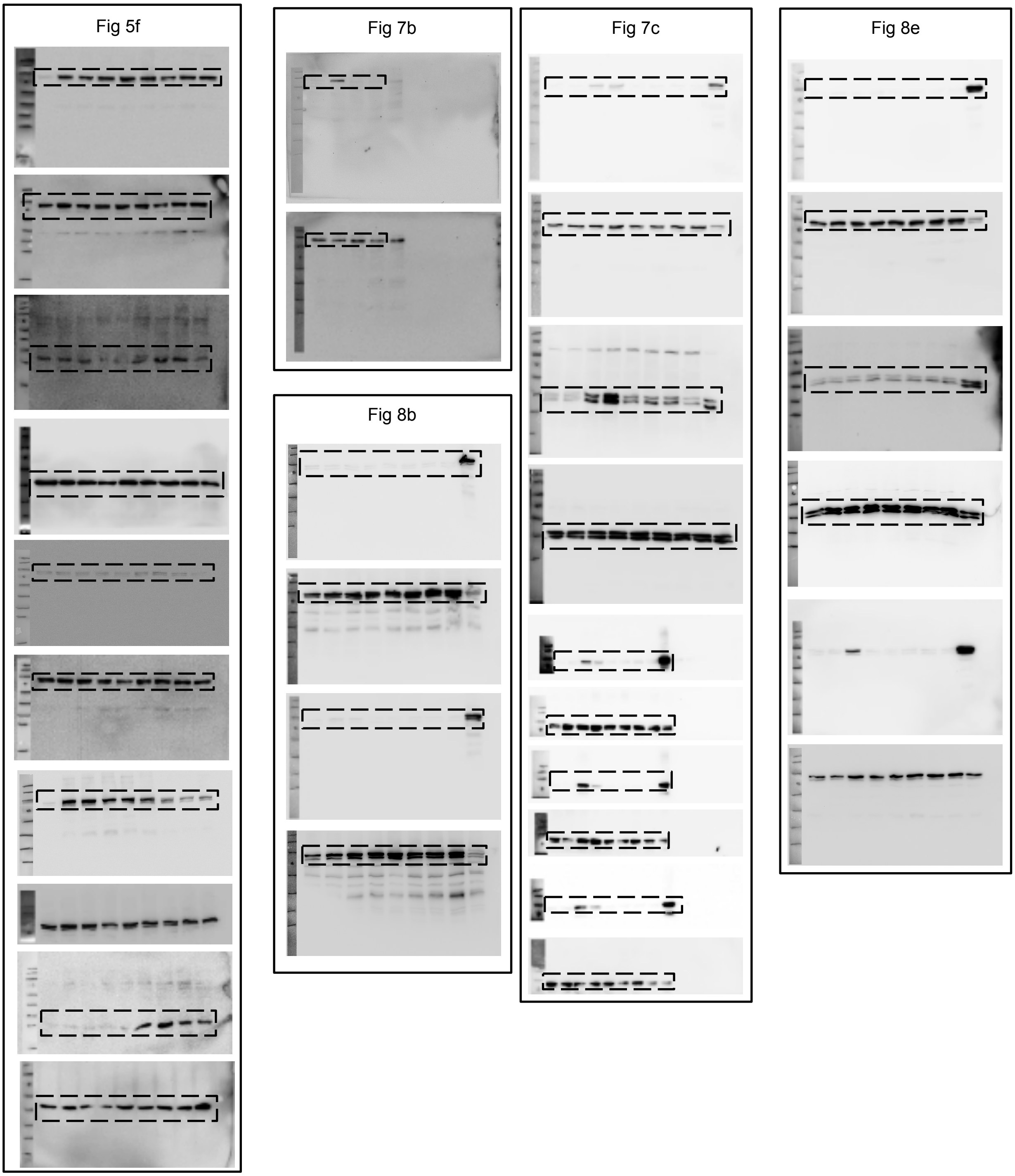

Supplementary Figure 13
Uncropped images of western blots presented in the main manuscript. Black dotted box indicates areas that were cropped.
